# Exploring the Phytochemical Profile and Therapeutic Potential of Saudi Native *Santolina chamaecyparissus* L. Essential Oil

**DOI:** 10.3390/pharmaceutics17070830

**Published:** 2025-06-26

**Authors:** Hanan Y. Aati, Wedad Sarawi, Hala Attia, Rehab Ghazwani, Lama Aldmaine

**Affiliations:** 1 Department of Pharmacognosy, College of Pharmacy, King Saud University, P.O. Box 2457, Riyadh 11451, Saudi Arabia; 2 Department of Pharmacology and Toxicology, College of Pharmacy, King Saud University, P.O. Box 2455, Riyadh 11451, Saudi Arabia; wsarawi@ksu.edu.sa (W.S.); hsalem@ksu.edu.sa (H.A.); 3 College of Pharmacy, King Saud University, P.O. Box 2457, Riyadh 11451, Saudi Arabia; 442200069@student.ksu.edu.sa (R.G.); 442201758@student.ksu.edu.sa (L.A.)

**Keywords:** *Santolina chamaecyparissus*, antioxidant, GC-MS, anti-inflammatory, cytotoxicity, molecular docking, ADMET, hemolytic, essential oil

## Abstract

**Background/Objectives:** Medicinal plants such as *Santolina chamaecyparissus* L., an evergreen shrub from the Asteraceae family, have long been valued for their bioactive compounds and traditional therapeutic uses. **Materials:** In this study, the essential oil of *S. chamaecyparissus* (EOSC) was isolated via hydrodistillation and then comprehensively evaluated for its phytochemical composition and antioxidant, anti-inflammatory, hemolytic, and cytotoxic properties, as well as its in silico bioactivity. **Results:** In total, 89.5% of the essential oil composition was successfully identified using GC-MS analysis. Hydrocarbon sesquiterpenes constituted the largest fraction (36.0%), followed by oxygenated sesquiterpenes (19.7%). Phytochemical screening revealed high phenolic content (839.50 ± 5.0 mg GAE/g E.O), while the Total Antioxidant Capacity (TAC) assay confirmed its strong antioxidant potential. The oil showed moderate hemolytic activity and significant lipoxygenase inhibition, indicating anti-inflammatory capability. The cytotoxic effects of the EOSC were evaluated using the MTT assay and HepG2 liver cancer cells. A dose-dependent reduction in cell viability was observed, confirming the oil’s strong anticancer activity. Molecular docking and ADMET analyses supported the bioactivity of the identified compounds, which showed good drug-likeness and pharmacokinetic properties. **Conclusions:** These findings demonstrate that EOSC has promising antioxidant and anti-inflammatory properties, suggesting that it could have potential as a safe natural substance for use in drug development and food preservation.

## 1. Introduction

Aromatic plants have long been valued for their therapeutic effects, primarily due to their essential oils, which are rich in diverse volatile compounds with well-established antimicrobial, antioxidant, and anti-inflammatory activities [1,2]. The genus Santolina (Asteraceae family) includes more than 10 widely distributed species. Many of these species have been studied for their biological and chemical properties, resulting in the identification of several mono- and sesquiterpenoids, as well as various other secondary metabolites [2]. *Santolina chamaecyparissus* L., widely recognized as cotton lavender, is a captivating small evergreen plant originating from the Mediterranean region, with additional ranges across Europe and America. This herb has gained attention due to its traditional use in European folk medicine for wound healing and treating digestive disorders and skin infections [3].

Recent phytochemical investigations have revealed that the essential oil of *S. chamaecyparissus* contains monoterpenes such as camphor, 1,8-cineole, borneol, and β-pinene, which are responsible for its pharmacological potential [1,3]. However, the variability in essential oil (EO) composition due to geographical and environmental factors necessitates continuous regional evaluation. For example, a study in Serbia identified significant regional differences in camphor and artemisia ketone concentrations [4].

Antioxidants play a crucial role in delaying or inhibiting the oxidation of lipids and other vital molecules. This is significant because the byproducts of lipid oxidation can adversely interact with biological materials, leading to cellular damage. Consequently, oxidation has been definitively linked to chronic diseases such as cancer. Embracing the use of medicinal plants could be a transformative step towards enhancing our health and preventing serious illnesses [5].

Lipoxygenases play a key role in the production of inflammatory mediators, and the inhibition of lipoxygenases, especially 5-lipoxygenase, can help to manage conditions such as asthma and arthritis. Many natural products and their secondary metabolites, such as flavonoids and terpenoids, have shown strong anti-inflammatory effects through the targeting of this enzyme, making them promising alternatives to conventional therapies [6,7].

The biological activities of EOSC, especially its antioxidant and enzyme inhibition effects, are of growing interest for potential therapeutics to manage oxidative stress-related diseases such as diabetes and neurodegenerative disorders [1,3]. However, the molecular mechanisms underlying these effects and their association with specific phytoconstituents remain underexplored.

In Saudi Arabia, *S. chamaecyparissus* is found in the southwestern region, Al-Jouf, Hail, and Al Madinah Al Munawarah. Despite its remarkable potential, research on the medicinal properties of this plant remains limited. To address this gap, the present study aimed to characterize the chemical composition of EOSC using GC-MS; assess its antioxidant and enzyme inhibition activities, and cytotoxic effects through various in vitro assays; and investigate its ligand–target interactions via molecular docking. This integrative approach provides a foundation for understanding the therapeutic potential of EOSC and supports its use in future drug discovery pipelines.

The results revealed strong therapeutic potential achieved by EOSC, suggesting its usefulness in treating oxidative stress, inflammation, and cancer. So, this essential oil could be used in medicine or in the development of plant-based nutraceuticals. Moreover, this study contributes to the chemotaxonomic understanding of Saudi medicinal flora and provides comparative insight into regional phytochemical variability.

## 2. Materials and Methods

### 2.1. Chemicals

The Folin–Ciocalteu reagent, gallic acid, anhydrous sodium carbonate, quercetin, sodium nitrile, ascorbic acid, a DPPH solution, ABTS, potassium persulfate, DMSO, sulfuric acid, sodium phosphate, ammonium molybdate, acetate buffer, ferric chloride, hydrochloric acid, 2,4,6-Tris (2-pyridyl)-s-triazine (TPTZ), sodium nitroprusside, sulfanilamide, phosphoric acid, naphthyl ethylenediamine dihydrochloride, sodium nitrile, sodium phosphate buffer, sodium chloride, di-nitro salicylic acid, streptokinase, chromogen, arachidonic acid, NDGA (nordihydroguaiaretic acid), a lipoxygenase solution, water, methanol, and ethanol utilized in this study were analytical grade and were obtained from Sigma Aldrich (St. Louis, MO, USA). All reference standards were also sourced from Sigma.

### 2.2. Preparation of Plant Essential Oil (EOSC)

In April 2024, approximately 800 g of fresh aerial parts of *S. chamaecyparissus* were collected during the flowering phase from Abha city in the southwestern part of Saudi Arabia (18°14′09.2″ N, 42°33′13.3″ E). The collection and handling of the plant material complied with the ethical standards and regulations established by King Saud University. The plant was taxonomically identified by Dr. Rajakrishnan Rajagopal, a botanist and plant taxonomist at the College of Science Herbarium, King Saud University, Riyadh. The authenticated specimen has been deposited in the university’s herbarium under the voucher reference number KSU-10682. The freshly collected plant material was stored in a refrigerator at 4 °C to preserve its phytochemical integrity. The time elapsed between collection and distillation was approximately 24 h. Essential oil extraction was subsequently carried out via the hydrodistillation (HD) technique using a Clevenger Apparatus. A total of 7 mL of the obtained essential oil (0.9% *w*/*v*), which exhibited an intense yellow-orange color, was stored in a glass vial protected from light in a refrigerator until the subsequent GC-MS phytochemical analysis and in vitro biological assays.

### 2.3. Phytochemical Analysis

#### 2.3.1. Total Bioactive Content

##### Total Phenolic Content (TPC)

The total phenolic content (TPC) was assessed using the Folin–Ciocalteu assay, following a previously established protocol [8,9]. A calibration curve was generated using gallic acid at various concentrations ranging from 5 to 50 µg/mL as the reference standard. The test oil was prepared at a concentration of 1 mg/mL, and a 125 µL aliquot was transferred into an Eppendorf tube. Subsequently, 500 µL of Folin–Ciocalteu reagent was added, followed by the addition of 375 µL of a 7.5% sodium carbonate (Na_2_CO_3_) solution. The mixture was then incubated in the dark for 2 h. After incubation, the absorbance was measured at 765 nm using a BioTek Synergy HT microplate reader (BioTek^®^, Boston, MA, USA). The TPC was expressed as milligrams of gallic acid equivalents per gram of essential oils (mg GAE/g E.O).

#### 2.3.2. Gas Chromatography–Mass Spectrometry Analysis (GC-MS)

An Agilent 5977A GC System with an HP-5MS capillary column measuring 30 m × 250 μm × 0.25 μm (with a maximum temperature of 350 °C) was used to profile the essential oil sample via GC-MS. This system was connected to an MSD system from the Agilent 5977A Series. The carrier gas was ultra-high-quality helium (99.99%) at a constant flow rate of 1.2 mL/min. Using an ionizing energy of 70 eV, the injection, transfer line, and ion source temperatures were all kept at 310 °C. The oven was set to rise at a rate of 5 °C per min from 60 °C (maintained for 7 min) to 310 °C. A 50:1 split ratio was used along with an injection volume of 1 μL. During the data-gathering process, full-scan mass spectra between 35 and 650 amu were recorded. The fragmentation patterns and the GC retention time were used to identify and classify the chemical substances. Using Mass Hunter GC/MS Acquisition, the mass spectra were compared to standards found in the NIST-02 mass spectrum library [10].

### 2.4. Biological Activities

#### 2.4.1. Antioxidant Activities

##### Measuring Total Antioxidant Capacity (TAC) Using Phosphomolybdenum Method

Total Antioxidant Capacity was measured using the protocol in [10] with minor modifications. Ascorbic acid was used as the standard; 1 mg of ascorbic acid was dissolved in 1 mL of 5% DMSO to create an ascorbic acid solution. Various ascorbic acid concentrations ranging from 50 to 1000 µg/mL were used. Standard test dilutions were made by adding 130 µL of the ascorbic standard solution to Eppendorf tubes and adjusting the final volume to 1 mL using the phosphomolybdenum reagent. For the test samples, 130 µL of the plant oil was combined with the phosphomolybdenum reagent to a final volume of 1 mL. The sample test solution and standard test dilutions were then incubated for 90 min at 95 °C in an incubator. The absorbance at 695 nm was measured.

##### 2,2-Diphenyl-1-picrylhydrazyl Assay (DPPH)

The test was performed according to the literature [10] with minor modifications. To make a DPPH solution, dissolve the DPPH in methanol until the concentration reaches 0.3 mM. In a microplate well, add an aliquot of the oil solution (90 µL) with the DPPH solution (90 µL). To allow the reaction to happen, incubate the mixture for a certain amount of time, typically 30 min, at room temperature in the dark. The absorbance of the resultant solution was measured with a BioTek Synergy HT microplate reader at 517 nm. The methanol was used as negative control, and the results were written as mg equivalent Ascorbic acid per gram of essential oil.

##### 2,2-Azino-bis(3-ethylbenzothiazoline-6-sulfonic Acid (ABTS) Assay

The ABTS assays were performed according to the method in [7] with some modifications. The reagent (2.5 mM ABTS + 2.45 mM K_2_S_2_O_8_ in 5% DMSO) was prepared and incubated at room temperature in the dark for 30 min to allow ABTS^+^ radical cations to form. A 90 µL volume of an ascorbic acid solution (1 mg/mL in 5% DMSO) was used to make a series of dilutions to draw a standard calibration curve. The calibration dilutions (5, 10, 15, 20, 25, 30, 40, 45, 50, 55, 60, 65, and 70 µg/mL) and oil (1 mg/mL in methanol) were added to the wells of a microplate. The prepared reagent was added to the wells, and the absorbance was measured at a wavelength of 515 nm using a BioTek Synergy HT (USA) microplate reader. The results were expressed as a mg equivalent of ascorbic acid per gram of essential oil.

##### Ferric Reducing Antioxidant Power (FRAP) Assay

The FRAP assay was performed according to the method in [11] with some modifications. The oil solution (1 mg/mL in methanol) was mixed with 90 µL of the reagent mixture [0.3 M acetate buffer (pH 3.6), 20 mM ferric chloride, and 10 mM 2,4,6-Tris (2-pyridyl)-s-triazine (TPTZ) in 40 mM HCl at a ratio of 10:1:1] in a microtiter plate and incubated for 30 min at room temperature. Then, the absorbance was measured at 593 nm using a BioTek Synergy HT (USA) microplate reader. The antioxidant potential was calculated based on the calibration curve. The results were expressed as a mg equivalent of ascorbic acid per gram of essential oil.

##### Nitric Oxide Scavenging (NOS) Assay

The Nitric Oxide Scavenging potential was determined by following a previously published method [10] with slight variations. Briefly, 1 mL of the oil solution (1 mg/mL) was mixed with 0.25 mL of a 25 mM sodium nitroprusside solution and incubated at 37 °C for 2 h. Then, 0.5 mL of the prepared solution was taken and mixed with 0.3 mL of Griess reagent (1% sulfanilamide, 2% phosphoric acid, and 0.1% naphthyl ethylenediamine dihydrochloride). The stock solution was sodium nitrite (1 mg/mL) in methanol, which was tested to draw a calibration curve. The absorbance was measured at 570 nm using a BioTek Synergy HT (USA) microplate reader.

#### 2.4.2. Enzyme Inhibition Activities

##### Lipoxygenase Inhibitory Activity

The anti-inflammatory activity of the plant oil was assessed in vitro using the Lipoxygenase Inhibitor Screening Assay, which was performed using the method described by Dilshad et al., 2023 [11], with slight modifications. An aliquot (10 μL) of the oil sample was added to 90 μL of the lipoxygenase enzyme solution. After allowing the mixture to incubate at room temperature for 5 min, arachidonic acid was added, and the plate was shaken for 10 min. Following this, 100 μL of chromogen was added, and the solution was agitated again for 5 min. The absorbance was measured at a wavelength of 500 nm. In the negative control, the plant oil was replaced with the assay buffer, while NDGA (nordihydroguaiaretic acid) was used as the positive control. The % inhibition was calculated as follows:

(%) Inhibition = (Absorbance of control − Absorbance of assay sample)/Absorbance of control × 100

#### 2.4.3. Hemolytic Activity

The hemolytic activity assay was conducted following previously published methods with slight modifications [12]. Human red blood cells (RBCs) were utilized to preliminarily evaluate the toxicity of the phytochemicals present in the plant oil. A total of 10 mL of human blood was collected from healthy volunteers into EDTA-containing tubes, which were then centrifuged at 4000 rpm for 10 min. The plasma layer was carefully removed, and the remaining RBCs were washed three times with phosphate-buffered saline (PBS) (pH 7.4). After washing, the RBCs were resuspended in PBS. For the assay, 975 µL of the oil solution (1 mg/mL in methanol) was mixed with 25 µL of the erythrocyte suspension in an Eppendorf tube. This mixture was incubated at 37 °C for 90 min, followed by centrifugation at 2000 rpm. The degree of hemolysis was determined by measuring the absorbance from hemoglobin released into the supernatant at 540 nm using a BioTek Synergy HT microplate reader. A 0.1% Triton X-100 solution served as the positive control, while PBS alone was used as the negative control. The percentage of hemolysis was calculated using the following formula:

Hemolysis (%) = (Absorbance of sample − Absorbance of negative control)/Absorbance of positive control × 100

#### 2.4.4. MTT Assay Using HepG2 (Liver Cancer) Cells

##### HepG2 Cell Line

The HepG2 liver cancer cell line was procured from the Animal Cell and Tissue Culture Laboratory at the Centre of Research in Molecular Medicine, Institute of Molecular Biology and Biotechnology, University of Lahore. The cells were stored in a liquid nitrogen tank and thawed from cryovials whenever culturing was required.

##### Culturing HepG2 Cell Line

The cells were revived and maintained in T75 culture flasks in high-glucose Dulbecco’s Modified Eagle’s Medium (DMEM) (Caisson’s Lab, Smithfield, UT, USA) supplemented with the antibiotics streptomycin and penicillin (Caisson’s Lab, USA) and 10% fetal bovine serum (FBS) (Sigma Aldrich, Burlington, MA, USA). Incubation was carried out in a humidified environment at 37 °C with 5% carbon dioxide. The culture medium was renewed every 2 to 3 days. For treatment procedures, DMEM without FBS was utilized.

##### Treatment of HepG2 Cell Line with EOSC

To allow for cell proliferation, HepG2 cells were cultured in 96-well plates. Some wells were left untreated to serve as controls, while the others were treated with different concentrations of the EOSC diluted in serum-free medium. The treated cells were exposed to the EOSC for 72 h. After the incubation period, the MTT assay was performed.

##### MTT Assay to Measure EOSC Cytotoxicity

Cell viability was determined using the MTT assay (Sigma Aldrich, USA). Each experimental group was tested in triplicate to ensure accuracy and reproducibility. To assess the proliferative response of HepG2 cells following treatment, the MTT assay was carried out in a 96-well plate. The cells were rinsed with phosphate-buffered saline (PBS) and incubated with 100 µL of serum-free DMEM along with 25 µL of an MTT solution (5 mg/mL) for 2 h. The resulting purple formazan crystals were dissolved in 10% sodium dodecyl sulfate (SDS), and the absorbance was measured at 570 nm. The percentage of viable cells was calculated following the method in [13]. The experiments were conducted in triplicate to determine the IC50 value. The following equation was used:

% Cell viability = Experimental (OD570)/Control (OD570) × 100

### 2.5. In Silico Studies

#### 2.5.1. Molecular Docking

Molecular docking is a useful approach in drug design and development. The structure of the intended protein was obtained from the Protein Data Bank (PDB) website (https://www.rcsb.org/) (accessed on 20 January 2025). The (PDB) IDs of lipoxygenase was 3o8y (resolution: 2.39 Ao). To prepare protein, Discovery Studio 2021 was used. Hetatoms and water molecules were eliminated followed by polar hydrogen addition. The final structures were then saved as (PDB) files. Bioactive compounds (ligands) obtained from GC-MS and standard compounds were downloaded from PubChem databases in SDF format. The Open Babel software converted ligands to PDB files. We used redocking and RMSD methodology for the validation of our protocol. The RMSD values were less than 2. Moreover, our docking scores for the standard inhibitor Quercetin (−9.1 kcal/mol) aligned well with its experimentally reported inhibitory activity, providing internal consistency to our approach. PyRx program, together with Autodock vina, was used for docking ligands to the active site of the specified enzymes. The exhaustiveness parameter which governs the extent of the search was selected as 8, and 9 modes were produced for every ligand. Based on the docking score, the optimal ligand orientation determination for the receptor was performed. To access the interactions that existed between the ligand–receptor complexes, the Discovery Studio Visualizer was employed [14].

We used redocking and RMSD methodology for the validation of our protocol. The RMSD values were less than 2. Moreover, our docking scores for the standard inhibitor Quercetin (−9.1 kcal/mol) aligned well with its experimentally reported inhibitory activity, providing internal consistency to our approach

#### 2.5.2. ADME Analysis

Using the online SwissADME tool http://www.swissadme.ch/ (accessed on 20 January 2025), the ADME properties of the bioactive compounds with the best molecular docking results were evaluated [15].

#### 2.5.3. Toxicity Evaluation

Using the online program PROTOX II https://tox-new.charite.de/ (accessed on 21 January 2025), the toxicity of the compounds with the best molecular docking results was evaluated.

## 3. Results

### 3.1. Phytochemical Analysis

#### 3.1.1. Total Phenolic Content (TPC)

The bioactive content analysis of the EOSC revealed significant amounts of phenolics (Table 1). The total phenolic content was 839.50 ± 5.0 mg GAE/g E.O, indicating a substantial presence of antioxidant, anti-inflammatory, and antimicrobial compounds. This finding suggests that the EOSC possesses a rich bioactive phenolic profile, making it a promising candidate for pharmaceutical or nutraceutical applications.

#### 3.1.2. GC-MS

**Figure 1:**
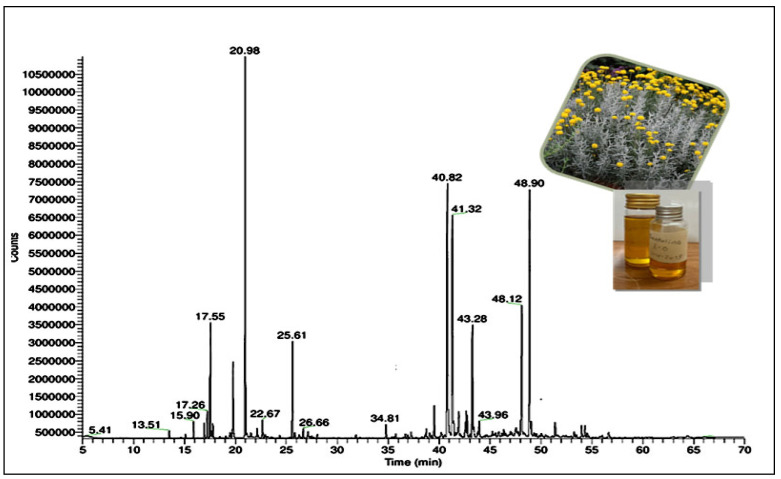
GC-MS chromatogram of EOSC.

The GC-MS chromatogram identified 50 distinct compounds in the essential oil, revealing a complex profile dominated by sesquiterpenes and monoterpenes in both hydrocarbon and oxygenated forms. The major constituents identified were artemisia ketone (15.5%), γ-curcumene (13.6%), α-bisabolol (11.3%), germacrene D (9.6%), vulgarone B (6.2%), trans-α-bisabolene (4.7%), myrcene (4.2%), camphor (3.8%), and β-phellandrene (3.0%), which, together, accounted for approximately 71.9% of the total oil content. In terms of chemical classification, hydrocarbon sesquiterpenes represented the highest proportion at 36.0%, followed by oxygenated sesquiterpenes (19.7%), hydrocarbon monoterpenes (11.0%), and oxygenated monoterpenes (6.0%). Other minor components comprised 16.8% of the oil. Overall, 89.5% of the total oil composition was identified, highlighting a rich mixture with potential pharmacological and therapeutic value due to the high content of bioactive terpenoids. These findings, which are detailed in Table 2, provide insights into the phytochemical composition of the EOSC; the chromatographic spectrum is shown in Figure 1.

### 3.2. Biological Activities of EOSC

#### 3.2.1. Antioxidant Activities

The antioxidant potential of the EOSC was evaluated using DPPH, FRAP, TAC, NOS, and ABTS assays. The results, presented in Table 3, highlight the correlation between antioxidant activity and bioactive content. The EOSC exhibited the highest activity in the TAC assay (1126.66 ± 0.66 mg eq. ascorbic acid/g E.O), indicating strong free-radical-scavenging potential. The DPPH assay revealed a maximum reduction potential of 234.50 ± 5.0 mg eq. ascorbic acid/g E.O, further confirming the EOSC’s antioxidant capability. The FRAP assay demonstrated an activity of 33.77±1.66 mg eq. ascorbic acid/g E.O, while the ABTS and NOS assays showed values of 72.83 ± 1.94 mg eq. ascorbic acid/g E.O and 16.72 ± 0.20 mg eq. ascorbic acid/g E.O, respectively. These findings suggest that the EOSC possesses significant antioxidant activity, making it a promising candidate for pharmaceutical and nutraceutical applications.

#### 3.2.2. Enzyme Inhibition Activities

The enzyme inhibition potential of the EOSC was assessed to explore its possible applications to treat inflammation through lipoxygenase inhibition. This study utilized quercetin as positive control. The lipoxygenase inhibition assay results demonstrated that the EOSC had an IC_50_ of 475.61 ± 0.19 µM, which was significantly different compared to that of quercetin (IC_50_ = 263.83 ± 0.22 µM) (Table 4), suggesting its potential in managing inflammation.

#### 3.2.3. Hemolytic Activity

**Figure 2:**
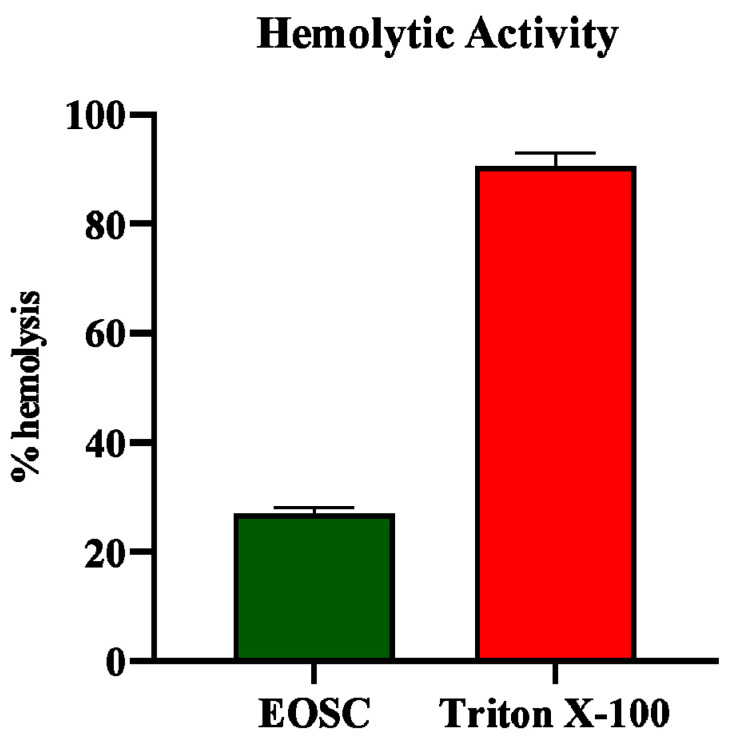
Hemolytic activity of EOSC.

The hemolytic activity of EOSC was evaluated, and the results are presented in Figure 2. The oil exhibited a hemolytic activity of 27.07 ± 0.95%**,** indicating a minimal hemolytic effect compared to the standard, which showed 90.57 ± 2.35% hemolysis. This low hemolytic activity suggests that EOSC has a favorable safety profile, making it a promising candidate for therapeutic applications with minimal cytotoxic effects on red blood cells.

#### 3.2.4. MTT Assay to Measure EOSC Cytotoxicity

**Figure 3:**
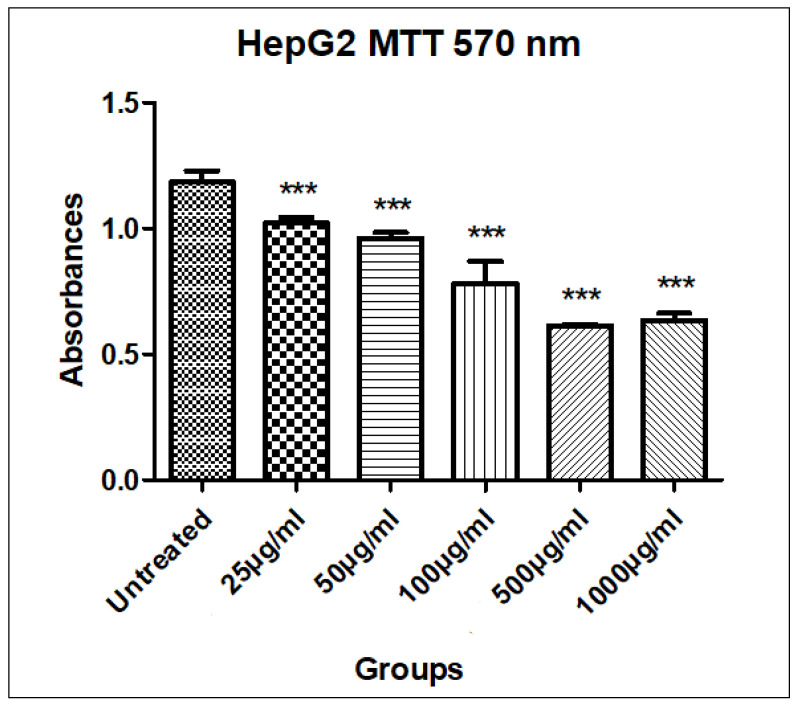
MTT assay of EOSC using different concentrations (25, 50, 100, 1000 µg/mL). Values taken were expressed as mean ± SD, ***: *p* < 0.001.

**Figure 4:**
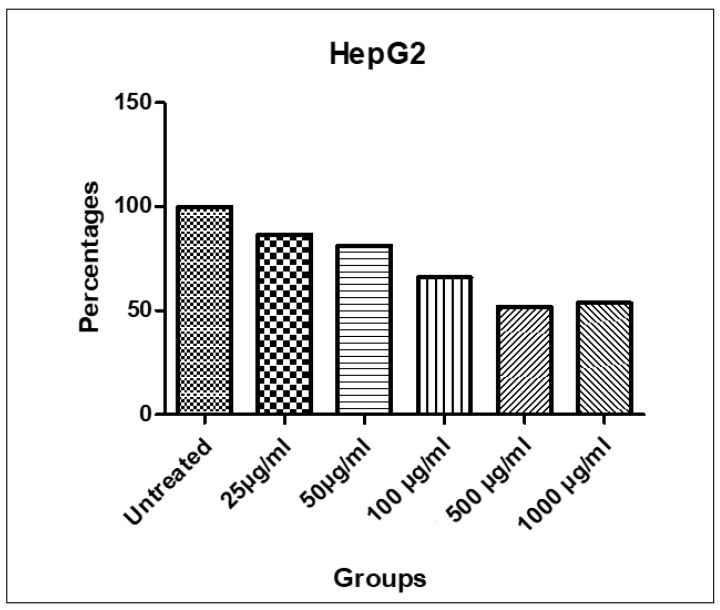
% Viability of HepG2 cells treated with 25, 50, 100, 500, and 1000 µg/mL of EOSC.

The MTT assay was performed to evaluate the cytotoxic potential of the EOSC against HepG2 liver cancer cells. The results revealed a promising dose-dependent reduction in cell viability. The untreated cells exhibited a mean absorbance of 1.18 ± 0.04, whereas the viability significantly decreased with increasing concentrations of the EOSC, indicating its potential anticancer efficacy. At the lowest tested concentration (25 µg/mL), the EOSC caused a slight decrease in viability (1.02 ± 0.02), suggesting limited cytotoxicity. However, a more pronounced effect was observed at 100 µg/mL (0.78 ± 0.09), and the maximum cytotoxicity was evident at 500 µg/mL (0.61 ± 0.01), followed by a slight increase at 1000 µg/mL (0.63 ± 0.03) (Table 5, Figure 3 and Figure 4). This pattern may indicate the saturation of the receptor or potential cytoprotective feedback mechanisms at very high concentrations.

### 3.3. In Silico Studies

#### 3.3.1. Molecular Docking

**Figure 5:**
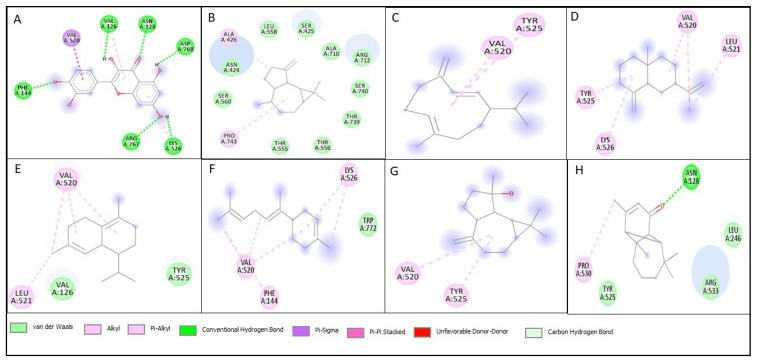
**Two-dimensional** structures of interaction of best docked compounds against lipoxygenase; (**A**) Quercetin (Standard), (**B**) Terpinolene, (**C**) Germacrene D, (**D**) β-Seliene, (**E**) δ-Cadinene, (**F**) trans-α-Bisabolene, (**G**) Spathulenol, (**H**) Vulgarone B.

Molecular docking studies were performed on all compounds identified by GC-MS against the lipoxygenase enzyme. The standard (Quercetin) for the lipoxygenase enzyme exhibited a binding affinity of −9.1 Kcal/mol, with no compounds showing higher binding affinity. The compounds with a relatively higher binding affinity than others are terpinolene, germacrene D, β-seliene, δ-cadinene, trans-α-bisabolene, spathulenol, and vulgarone B. The key interactions observed in these compounds include conventional hydrogen bonds, Pi-sigma, Pi-Pi stacked, Pi-alkyl, van der Waals forces, alkyl interactions, unfavorable donor–donor and carbon–hydrogen bonds (Table 6, Figure 5).

#### 3.3.2. ADME Analysis

**Figure 6:**
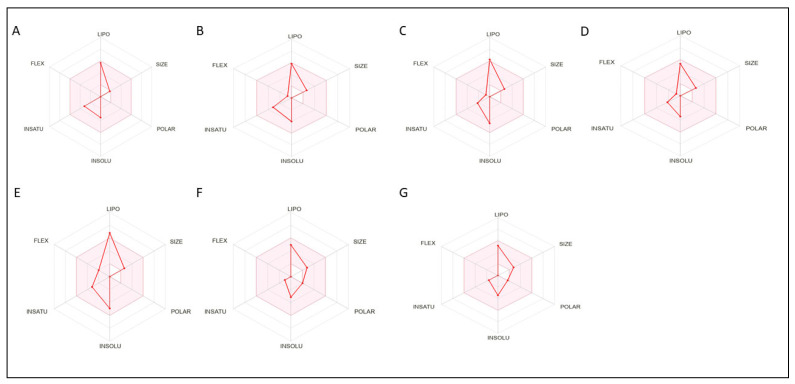
Bioavailability radar of phytocompounds showing best binding affinities; (**A**) Terpinolene, (**B**) Germacrene D, (**C**) β-Seliene, (**D**) δ-Cadinene, (**E**) trans-α-Bisabolene, (**F**) Spathulenol, (**G**) Vulgarone B.

Our study’s findings indicate that the ADME analysis of selected phytoconstituents from EOSC revealed that only one compound showed no violations, while four compounds exhibited two violations, and two compounds exhibited one violation, as presented in Table 7. Figure 6 illustrates the bioavailability radar of the selected phytoconstituents from EOSC. Furthermore, no previous studies have reported the ADME analysis of EOSC in the literature. Therefore, we were the first to study the ADME profile of EOSC and to present its results in this study.

#### 3.3.3. Toxicity Analysis

The phytoconstituents of the EOSC with the highest binding affinity (best docking scores) were assessed for toxicity using the PROTOX online tool. This software provides insights into the predicted LD50, toxicity classification, hepatotoxicity, carcinogenicity, mutagenicity, immunotoxicity, and cytotoxicity of compounds. The toxicity analysis revealed that all the phytoconstituents tested negative for hepatotoxicity, carcinogenicity, mutagenicity, immunotoxicity, and cytotoxicity. However, germacrene D exhibited immunotoxic potential. All the phytoconstituents were classified as toxicity class 5. The detailed toxicity evaluation results are presented in Table 8.

## 4. Discussion

Natural products have played a crucial role in the development of remedies for many contemporary diseases. They are a rich source of bioactive compounds with diverse therapeutic potential, including anti-inflammatory, antioxidant, antimicrobial, and anticancer substances. These naturally derived substances often have better safety profiles and fewer side effects compared to synthetic drugs [3]. Using remedies derived from natural sources can offer multiple benefits: they are often more biocompatible, biodegradable, and culturally accepted, especially in regions with a strong traditional use of herbal medicine. In addition, natural product-based therapies often support holistic healing, addressing not just the symptoms but the underlying imbalances in the body.

Aromatic plants are highly valued for their fragrant essential oils, which contain numerous active compounds with therapeutic properties. These plants are widely used in traditional medicine, aromatherapy, cosmetics, and the food industry. Their antimicrobial and mood-enhancing effects are attracting growing attention in both modern and alternative medicine [2]. One of the most important plant families with aromatic and medicinal properties is the Asteraceae family. It is one of the largest families of flowering plants, known for their wide distribution and high medicinal value. Many members of this family are rich in essential oils and secondary metabolites that exhibit a range of biological activities and are used globally in traditional and modern medicine for treating infections, inflammation, and digestive disorders [2,3].

Among the valuable plants in this family is *S. chamaecyparissus*, a perennial aromatic shrub traditionally used for its medicinal properties. Despite its potential, studies on this plant remain limited, especially studies on its biological activities and chemical composition in various geographic regions. In the Kingdom of Saudi Arabia, which is known for its rich biodiversity and variety of medicinal plants due to its diverse climate and terrain, *S. chamaecyparissus* can be found in certain areas [3]. It has been used traditionally for treating ailments such as digestive problems and skin conditions, yet scientific research on the local varieties is limited.

Essential oils, as concentrated extracts of aromatic plants, are known for their potent biological effects. They exhibit a range of therapeutic properties such as antimicrobial, antifungal, antiviral, and anti-inflammatory actions. These oils have found applications not only in traditional medicine but also in pharmaceuticals, cosmetics, and natural food preservation. The essential oil of *S. chamaecyparissus* showed promising antimicrobial and antioxidant activities in initial studies [2,3,4]. However, its chemical profile can vary greatly depending on environmental and geographic factors, and research in Saudi Arabia is still in its early stages. Previous investigations into the essential oil composition and biology of *S. chamaecyparissus* in Saudi Arabia are limited in number and scope, often lacking comprehensive phytochemical analyses or robust biological evaluations.

This study revealed that the total phenolic content of the EOSC was high (839.50 ± 5.0), which suggests that phenols are the dominant bioactive compounds in this EOSC. Phenolic compounds play a crucial role in enhancing plants’ quality and nutritional value by influencing their color, taste, aroma, and flavor while delivering significant health benefits. Additionally, they are vital for plant defense mechanisms, effectively combating reactive oxygen species (ROS) to ensure survival and mitigate molecular damage caused by microorganisms, insects, and herbivores [16]. Phenolic compounds are essential in safeguarding cells against oxidative damage through their antioxidant capabilities [17]. These powerful polyphenolic compounds can scavenge free radicals and inhibit harmful hydrolytic and oxidative enzymes, and they exhibit strong anti-inflammatory effects. Phenolics can be isolated from a variety of plants, and compounds like flavonoids are a vital component of a healthy diet, playing a crucial role in enhancing our body’s defense systems and promoting overall well-being [18]. Studies have shown that plant phenolics have antioxidant, anti-inflammatory, and anticancer activities [3]. This highlights the ecological significance of these compounds in plant adaptation and their potential pharmacological benefits.

A GC-MS analysis was conducted to identify the phytochemicals in the EOSC. The GC-MS chromatogram identified 50 distinct compounds in the essential oil; 89.5% of the essential oil’s composition was successfully determined, revealing a diverse array of bioactive terpenoids with notable therapeutic potential. The oil was dominated by sesquiterpenes and monoterpenes in both hydrocarbons and oxygenated forms. Hydrocarbon sesquiterpenes emerged as the dominant class, comprising 36.0% of the oil, followed by oxygenated sesquiterpenes at 19.7%, hydrocarbon monoterpenes at 11.0%, and oxygenated monoterpenes at 6.0%. The remaining 16.8% consisted of various other constituents. The major constituents identified were artemisia ketone (15.5%), γ-curcumene (13.6%), α-bisabolol (11.3%), germacrene D (9.6%), vulgarone B (6.2%), myrcene (4.2%), camphor (3.8%), and β-phellandrene (3.0%), which, together, accounted for approximately 67.2% of the total oil content. Among these compounds, α-bisabolol and myrcene have substantial evidence supporting their antioxidant, anti-inflammatory, and cytotoxic activities, including effects on liver cancer cells [19,20].

Terpenoids possess a diverse range of medicinal properties, including antioxidant, anticancer, antiseptic, anti-inflammatory, antiplasmodial, digestion-related, astringent, and diuretic activities [21]. Due to the presence of these bioactive phytochemicals, EOSC appears to be a promising candidate for pharmaceutical applications because it contains a variety of terpenoid constituents which might work alone or synergistically to produce interesting biological effects.

Moreover, the EOSC exhibited a remarkable level of lipoxygenase inhibition activity, underscoring its significant potential in combating inflammation. Lipoxygenases and their derived products are intricately linked to various carcinogenic processes, including tumor cell proliferation, differentiation, and apoptosis. This versatile class of oxidative enzymes plays a critical role in the metabolism of arachidonic acid. Recently, a growing array of arachidonic acid metabolites have been uncovered, with well-established pro-inflammatory effects and emerging anti-inflammatory properties [22].

Hemolysis—the breakdown of red blood cells (RBCs)—is a crucial biological process. This breakdown leads to the release of hemoglobin into plasma, significantly altering its color. Understanding hemolysis is essential, as it plays a vital role in various medical conditions and diagnostics [23]. Certain plant extracts pose a risk of disrupting red blood cell membranes, which can lead to serious health issues such as hemolytic anemia. Therefore, it is vital to evaluate the hemolytic activity of these extracts to ensure their safety for human use. Thorough assessment is essential to safeguard public health and prevent potential adverse effects. In our study, the EOSC was found to exhibit a hemolysis rate of 27.07 ± 0.95% (Figure 2), which is below the 30% toxicity threshold, confirming that the extract is non-toxic and safe for human consumption. To our knowledge, no previous research has reported hemolytic effects for EOSC, making this the first study to evaluate its safety in this regard.

To evaluate the enzyme inhibition potential of the compounds identified in the GC-MS analysis of the EOSC, molecular docking studies were conducted on lipoxygenase. This analysis aimed to determine the compounds’ binding affinities and establish a correlation between in vitro enzyme inhibition and computational predictions. The standard inhibitor quercetin exhibited a score of −9.1 Kcal/mol. Among the EOSC compounds, trans-α-bisabolene was the most active, with a binding affinity of −7.8 Kcal/mol. It effectively interacted within the active site of lipoxygenase, forming van der Waals interactions, Alkyl, and Pi-Alkyl bonds with key amino acid residues. In contrast, Terpinolene, Germacrene D, β-Seliene, δ-Cadinene, Spathulenol, and Vulgarone B primarily exhibited conventional hydrogen bond interactions, van der Waals interactions, Alkyl, Pi-Alkyl bonds, Pi-sigma, Pi-Pi stacked, and carbon hydrogen bonds. Overall, the docking studies provided valuable insights into the mechanisms of enzyme inhibition, supporting the in vitro findings and highlighting EOSC as a promising source of bioactive compounds.

The dose-dependent cytotoxicity of EOSC aligns with previous findings revealing that essential oils from medicinal plants can disrupt cancer cell metabolism through apoptosis induction, membrane disruption, and modulation of signaling pathways [24]. Specifically, *S. chamaecyparissus* has been reported to contain bioactive compounds such as camphor, 1,8-cineole, and borneol [25], which are known for their antioxidant and antiproliferative properties. These findings support the traditional medicinal use of EOSC and underscore its potential as a source of natural anticancer agents. However, further investigations, including apoptosis assays, ROS measurement, and in vivo models, are essential to elucidate its exact mechanisms and therapeutic potential.

To further evaluate the drug-like properties of the compounds with the highest affinities (best docking scores), we conducted an ADME analysis using the “SwissADME” web application to gain insights into the pharmacokinetics, physicochemical properties, and drug-likeness attributes of seven bioactive phytoconstituents. According to Lipinski’s Rule of Five, a compound is considered a poor candidate for oral administration if it violates two or more rules (molecular weight, lipophilicity (LogP), hydrogen bond donors, molar refractivity, and hydrogen bond acceptors) [26]. Our analysis revealed that all the compounds fully complied with Lipinski’s Rule of Five with no violations, suggesting that they have high drug-likeness for oral use. Only one compound exhibited one violation, indicating moderate potential for oral drug development. Table 7 and Figure 6 present the bioavailability radar charts of the selected phytoconstituents, which illustrate the key parameters: lipophilicity, pharmacokinetics, molecular flexibility (bond rotations), molecular weight, and hydrogen bond interactions. These properties play a crucial role in determining drug absorption and efficacy in the human body and provide a detailed breakdown of the pharmacokinetic behavior and physicochemical properties of these compounds. To our knowledge, no prior studies have performed an ADME analysis of EOSC. Our findings highlight its potential for oral drug formulations, offering advantages such as improved safety, patient compliance, and ease of administration compared to other drug delivery methods. These results support the further exploration of EOSC phytoconstituents for therapeutic applications.

To assess the toxicity profile of the seven EOSC phytoconstituents with the highest docking scores, we utilized the ProTox-II online tool. This program predicts toxicity levels by comparing the chemical structures of the selected compounds to known toxic substances [26]. The analysis provided predicted LD50 values, toxicity classifications, and potential hepatotoxicity, carcinogenicity, mutagenicity, immunotoxicity, and cytotoxicity data. All the phytoconstituents tested negative, indicating they are unlikely to cause liver damage, genetic mutations, or cytotoxic effects. Germacrene D was predicted to exhibit immunotoxicity effects, implying possible alterations to immune system modulation. The phytoconstituents fell into toxicity class 5, indicating a moderate to low toxicity range, which suggests that they are relatively safe for therapeutic applications. To our knowledge, no prior studies have performed toxicity analyses of EOSC. These findings provide critical safety data for the further exploration of EOSC-derived bioactive compounds for pharmaceutical applications. Future studies should include in vivo validation and dose-dependent toxicity assessments to confirm the safety and therapeutic potential of these phytoconstituents.

Several studies have investigated the essential oil of *S. chamaecyparissus* from different regions, with a focus on its phytochemical composition and biological activities. In a study in Tunisia, GC and GC-MS analyses identified 67 constituents, which accounted for 99.14% and 99.44% of the total flowerhead and root oils, respectively, with 1,8-cineole (12.94%) and β-eudesmol (10.49%) being among the major compounds. The oils demonstrated potent antibacterial activity—particularly against *P. aeruginosa* and *E. faecalis*—and showed significant antifungal effects against dermatophytes and other fungi [2]. In Saudi Arabia, one study investigated the seasonal variations in the oil composition and found 39 key compounds, including curcumene, α-terpinol, p-cymene, 1,8-cineole, and caryophyllene oxide. The oil exhibited notable cytotoxicity against HepG2 liver cancer cells, achieving 97.7% inhibition, although it showed limited activity against other cancer cell lines [1].

In a study in India, an ethyl acetate extract of *S. chamaecyparissus* leaves was examined for its antidiabetic and anticancer properties. The GC-MS analysis revealed 44 compounds, with tetrapentacontane (27.15%) and eicosyl acetate (8.40%) being the most abundant. The extract moderately inhibited α-glucosidase (IC_50_ = 110 ± 4.25 µg/mL), suggesting that it has antidiabetic activity, and downregulated Epidermal Growth Factor Receptor (EGFR) expression in a human breast cancer cell line (MCF7), suggesting that it has anticancer effects [27]. Similarly, in a study in Algeria, EOSC showed strong antioxidant and antimicrobial effects, validating its traditional medicinal uses. The GC-MS analysis identified a total of 36 compounds, accounting for 91.7% of the essential oil; in particular, camphor (31.1%) and cubenol (17.0%) were the predominant compounds [28]. A Greek study identified 54 oil constituents through GC-MS, including artemisia ketone, vulgarone B, and a unique spiroketal-enol ether polyynic compound, highlighting the chemical richness of the species [29].

In a study in Turkey, Santolina essential oil was found to contain high levels of artemisia ketone (39.83%) and camphor (17.65%). It showed strong antioxidant activity and inhibited the growth of various microorganisms, such as *E. coli* and *C. albicans* [30]. Additionally, another Algerian study isolated luteolin-7-O-glucoside from the leaf extract which, along with the crude polyphenolic extract, exhibited significant antioxidant properties, further supporting its potential as a natural antioxidant source [31]. Previous studies on EOSC have shown variations in its components, even though it is the same species. This highlights the influence of environmental factors, climate, and geographical location on the diversity and proportion of its constituents, which, in turn, affects its biological activity. This emphasizes the importance of the current study not only in revealing the biological significance of the oil but also in highlighting the necessity of analyzing the chemical composition of the Saudi species, which can serve as a chemotaxonomic tool for *S. chamaecyparissus* classification.

In summary, the essential oil of *S. chamaecyparissus* contains a diverse array of bioactive compounds, particularly terpenoids, with proven antimicrobial, antioxidant, antidiabetic, and anticancer properties. While studies from Tunisia, India, Algeria, Greece, and Turkey have provided valuable insights, investigations specific to the Saudi Arabian species remain limited in number and scope. This highlights the need for further research to explore the unique chemical composition and therapeutic potential of *S. chamaecyparissus* grown in Saudi Arabia. For these reasons, we conducted this study to address the current gaps in our knowledge on *S. chamaecyparissus* grown in Saudi Arabia. Through studying its essential oil composition and evaluating its biological potential, our work provides a scientific foundation for future therapeutic applications and contributes to our understanding of this underutilized aromatic species.

## 5. Conclusions

The present study comprehensively evaluated the phytochemical composition, biological activities, enzyme inhibition potential, drug-likeness, and toxicity profile of EOSC. The GC-MS analysis identified 50 bioactive compounds. The antioxidant assays revealed that EOSC has significant free radical-scavenging activity. Moreover, the EOSC exhibited notable lipoxygenase inhibition, underscoring its potential in combating inflammation. Its hemolytic activity was below the toxicity threshold, indicating that it is safe for therapeutic use. Importantly, the MTT assay demonstrated that the EOSC exhibited dose-dependent cytotoxicity against HepG2 liver cancer cells, with the maximum activity observed at a concentration of 500 µg/mL. This highlights its anti-cancer potential and supports its use in oncological research. The ADME analysis showed that most of the tested compounds adhered to Lipinski’s Rule of Five, suggesting good oral bioavailability. The toxicity predictions indicated moderate to low toxicity, with no hepatotoxic, mutagenic, or cytotoxic effects observed in silico. To the best of our knowledge, this is the first comprehensive investigation of Saudi EOSC, which demonstrated EOSC’s pharmaceutical potential. Future studies should focus on in vivo validation, formulation development, and mechanistic explorations to verify its clinical relevance.

## Figures and Tables

**Table 1:** Total phenolic content of EOSC.

Sample	TPC (mg GAE/g E.O)
EOSC	839.50 ± 5.0

All the assays were performed in triplicate, and the results are expressed as the mean ± standard deviation.

**Table 2:** Phytocompounds identified in the GC-MS of EOSC; the major constituents are highlighted in bold.

#	Retention Time	% Area	Compound Name
1.	13.51	0.3	Santolinatriene
2.	15.12	0.2	α-Pinene
3.	15.90	0.6	Camphene
4.	16.96	0.5	Sabinene
5.	17.26	1.0	β-pinene
6.	17.55	4.2	**Myrcene**
7.	17.79	0.6	Yomogi alcohol
8.	18.48	0.1	α-Phellandrene
9.	19.08	0.1	α-Terpinene
10.	19.42	0.1	p-Cymene
11.	19.70	0.2	Limonene
12.	19.78	3.0	**β-Phellandrene**
13.	20.98	15.5	**Artemisia ketone**
14.	21.14	0.2	γ-Terpinene
15.	22.15	0.4	Artemisia alcohol
16.	22.67	0.7	Terpinolene
17.	25.38	0.1	trans-Pinocarveol
18.	25.61	3.8	**Camphor**
19.	25.85	0.2	Chrysanthemol
20.	26.27	0.2	Lavandulol
21.	26.66	0.4	Borneol
22.	27.15	0.3	Terpinen-4-ol
23.	27.51	0.1	Cryptone
24.	28.06	0.2	Myrtenal
25.	34.81	0.6	δ-Elemene
26.	35.74	0.2	α-Longipinene
27.	36.71	0.2	α-Copaene
28.	38.65	0.1	β-Ylangene
29.	38.77	0.4	β-Caryophyllene
30.	39.09	0.3	β-Copaene
31.	39.28	0.1	Sesquisabinene B
32.	39.54	1.4	(E)-β-Farnesene
33.	40.25	0.2	α-Caryophyllene
34.	40.82	13.6	**γ** **-Curcumene**
35.	41.32	9.6	**Germacrene D**
36.	41.48	0.5	γ-Humulene
37.	41.60	0.2	β-Seliene
38.	41.94	1.4	Bicyclogermacrene
39.	42.60	0.3	γ-Cadinene
40.	42.65	1.2	Sesquiphellandrene
41.	42.78	1.0	δ-Cadinene
42.	43.03	0.1	Isohumbertiol B
43.	43.28	4.7	**trans-α-Bisabolene**
44.	43.96	0.7	(E)-Nerolidol
45.	45.29	0.2	Spathulenol
46.	45.56	0.2	Caryophyllene oxide
47.	47.57	0.6	τ-Muurolol
48.	46.63	1.2	Dillapiole
49.	48.12	6.2	**Vulgarone B**
50.	48.90	11.3	**α-Bisabolol**
**Oxygenated monoterpenes**		6.0%	
**Hydrocarbon monoterpenes**		11.0%	
**Oxygenated sesquiterpenes**		19.7%	
**Hydrocarbon sesquiterpenes**		36.0%	
**Other**		16.8%	
**Total %**		**89.5%**	

**Table 3:** Antioxidant potential of the EOSC.

Sample	ABTS	DPPH	FRAP	TAC	NOS
EOSC	72.83 ± 1.94	234.50 ± 5.0	33.77 ± 1.66	1126.66 ± 0.66	16.72 ± 0.20

All the assays were performed in triplicate. The results were calculated as mg ascorbic acid/g E.O and are expressed as the mean ± standard deviation.

**Table 4:** Lipoxygenase inhibition potential of EOSC.

Sample	Lipoxygenase Enzyme Inhibition IC_50_ µM
EOSC	475.61 ± 0.19
Quercetin	263.83 ± 0.22

**Table 5:** The cell viability of EOSC on HepG2 cells.

Group	Cell Viability Value (Mean ± SD)
Untreated	1.18 ± 0.04
Treated with EOSC (25 µg/mL)	1.02 ± 0.02
Treated with EOSC (50 µg/mL)	0.96 ± 0.02
Treated with EOSC (100 µg/mL)	0.78 ± 0.09
Treated with EOSC (500 µg/mL)	0.61 ± 0.01
Treated with EOSC (1000 µg/mL)	0.63 ± 0.03

**Table 6:** Binding affinity of GC-MS phytocompounds against lipoxygenase enzyme (the top compounds with an affinity ≥ −7.5 are highlighted in bold).

#	Compound Name	Binding Affinity for Lipoxygenase (Kcal/mol)	#	Compound Name	Binding Affinity for Lipoxygenase (Kcal/mol)
1.	Santolina triene	−5.0	26.	α-Longipinene	−7.1
2.	α-Pinene	−6.4	27.	α-Copaene	−7.2
3.	Camphene	−5.8	28.	β-Ylangene	−7.3
4.	Sabinene	−5.7	29.	β-Caryophyllene	−7.0
5.	β-pinene	−6.3	30.	β-Copaene	−7.3
6.	Myrcene	−5.5	31.	Sesquisabinene B	−6.1
7.	Yomogi alcohol	−5.4	32.	(E)-β-Farnesene	−5.9
8.	α-Phellandrene	−6.1	33.	α-Caryophyllene	−7.2
9.	α-Terpinene	−5.9	34.	γ-Curcumene	−7.4
10.	p-Cymene	−6.0	35.	**Germacrene D**	**−7.5**
11.	Limonene	−5.9	36.	γ-Humulene	−7.2
12.	β-Phellandrene	−5.9	37.	**β-Seliene**	**−7.7**
13.	Artemisia ketone	−5.2	38.	Bicyclogermacrene	−7.4
14.	γ-Terpinene	−6.0	39.	γ-Cadinene	−6.9
15.	Artemisia alcohol	−5.1	40.	Sesquiphellandrene	−5.7
16.	**Terpinolene**	**−7.6**	41.	**δ-Cadinene**	**−7.7**
17.	trans-Pinocarveol	−5.9	42.	Isohumbertiol B	−6.1
18.	Camphor	−5.9	43.	**trans-α-Bisabolene**	**−7.8**
19.	Chrysanthemol	−5.4	44.	(E)-Nerolidol	−6.1
20.	Lavandulol	−5.2	45.	**Spathulenol**	−**7.5**
21.	Borneol	−5.5	46.	Caryophyllene oxide	−7.3
22.	Terpinen-4-ol	−6.1	47.	τ-Muurolol	−7.2
23.	Cryptone	−5.6	48.	Dillapiole	−6.2
24.	Myrtenal	−5.8	49	**Vulgarone B**	**−7.5**
25.	δ-Elemene	−6.8	50.	α-Bisabolol	−7.3
**Standard (Quercetin)**			**−9.1**		

**Table 7:** Physicochemical properties and Lipinski’s rule of best docked compounds.

#	Compound Name	HBA	HBD	MWT	Lipophilicity	M.R	LR
1.	Terpinolene	0	0	204.35	5.65	67.14	Yes; 1 violation
2.	Germacrene D	0	0	204.35	4.53	70.68	Yes; 0 violations
3.	β-Seliene	0	0	204.35	4.63	68.78	Yes; 0 violations
4.	δ-Cadinene	0	0	204.35	4.63	69.04	Yes; 0 violations
5.	trans-α-Bisabolene	0	0	204.35	4.53	70.68	Yes; 0 violations
6.	Spathulenol	1	1	220.35	3.67	68.34	Yes; 0 violations
7.	Vulgarone B	1	0	218.33	3.56	67.08	Yes; 0 violations

HBA, hydrogen bond acceptor; HBD, hydrogen bond donor; M.R, molar refractivity; MWT, molecular weight.

**Table 8:** Predicted toxicity evaluation of EOSC.

#	Compound	Predicted LD_50_ (mg/kg)	Predicted Toxicity Class	Hepatotoxicity	Carcinogenicity	Mutagenicity	Immunotoxicity	Cytotoxicity
1.	Terpinolene	5000	5	**−**	**−**	**−**	**−**	**−**
2.	Germacrene D	5300	5	**−**	**−**	**−**	**+**	**−**
3.	β-Seliene	5000	5	**−**	**−**	**−**	**−**	**−**
4.	δ-Cadinene	4390	5	**−**	**−**	**−**	**−**	**−**
5.	trans-α-Bisabolene	4390	5	**−**	**−**	**−**	**−**	**−**
6.	Spathulenol	3900	5	**−**	**−**	**−**	**−**	**−**
7.	Vulgarone B	2300	5	**−**	**−**	**−**	**−**	**−**

(**+**): toxic; (**−**): not toxic.

## Data Availability

Data are contained within the article.
